# Genome-wide association study and RNA-seq identifies GmWRI1-like transcription factor related to the seed weight in soybean

**DOI:** 10.3389/fpls.2023.1268511

**Published:** 2023-11-17

**Authors:** Qin Di, Lidong Dong, Li Jiang, Xiaoyi Liu, Ping Cheng, Baohui Liu, Guohui Yu

**Affiliations:** ^1^Innovative Institute for Plant Health, Zhongkai University of Agriculture and Engineering, Guangzhou, China; ^2^Innovative Center of Molecular Genetics and Evolution, College of Life Sciences, Guangzhou University, Guangzhou, Guangdong, China; ^3^Research Center of Integrative Medicine, School of Basic Medical Sciences, Guangzhou University of Chinese Medicine, Guangzhou, Guangdong, China

**Keywords:** soybean, seed weight, oil content, GWAS, candidate gene

## Abstract

The cultivated soybean (*Glycine max* (L.) Merrill) is domesticated from wild soybean (*Glycine soja*) and has heavier seeds with a higher oil content than the wild soybean. In this study, we identified a novel candidate gene associated with SW using a genome-wide association study (GWAS). The candidate gene *GmWRI14*-like was detected by GWAS analysis in three consecutive years. By constructing transgenic soybeans overexpressing the *GmWRI14-*like gene and *gmwri14-*like soybean mutants, we found that overexpression of *GmWRI14-*like increased the SW and increased total fatty acid content. We then used RNA-seq and qRT-PCR to identify the target genes directly or indirectly regulated by *GmWRI14-*like. Transgenic soyabeans overexpressing *GmWRI14-*like showed increased accumulation of *GmCYP78A50* and *GmCYP78A69* than non-transgenic soybean lines. Interestingly, we also found that GmWRI14-like proteins could interact with GmCYP78A69/GmCYP78A50 using yeast two-hybrid and bimolecular fluorescence complementation. Our results not only shed light on the genetic architecture of cultivated soybean SW, but also lays a theoretical foundation for improving the SW and oil content of soybeans.

## 1 Introduction

**Figure 1:**
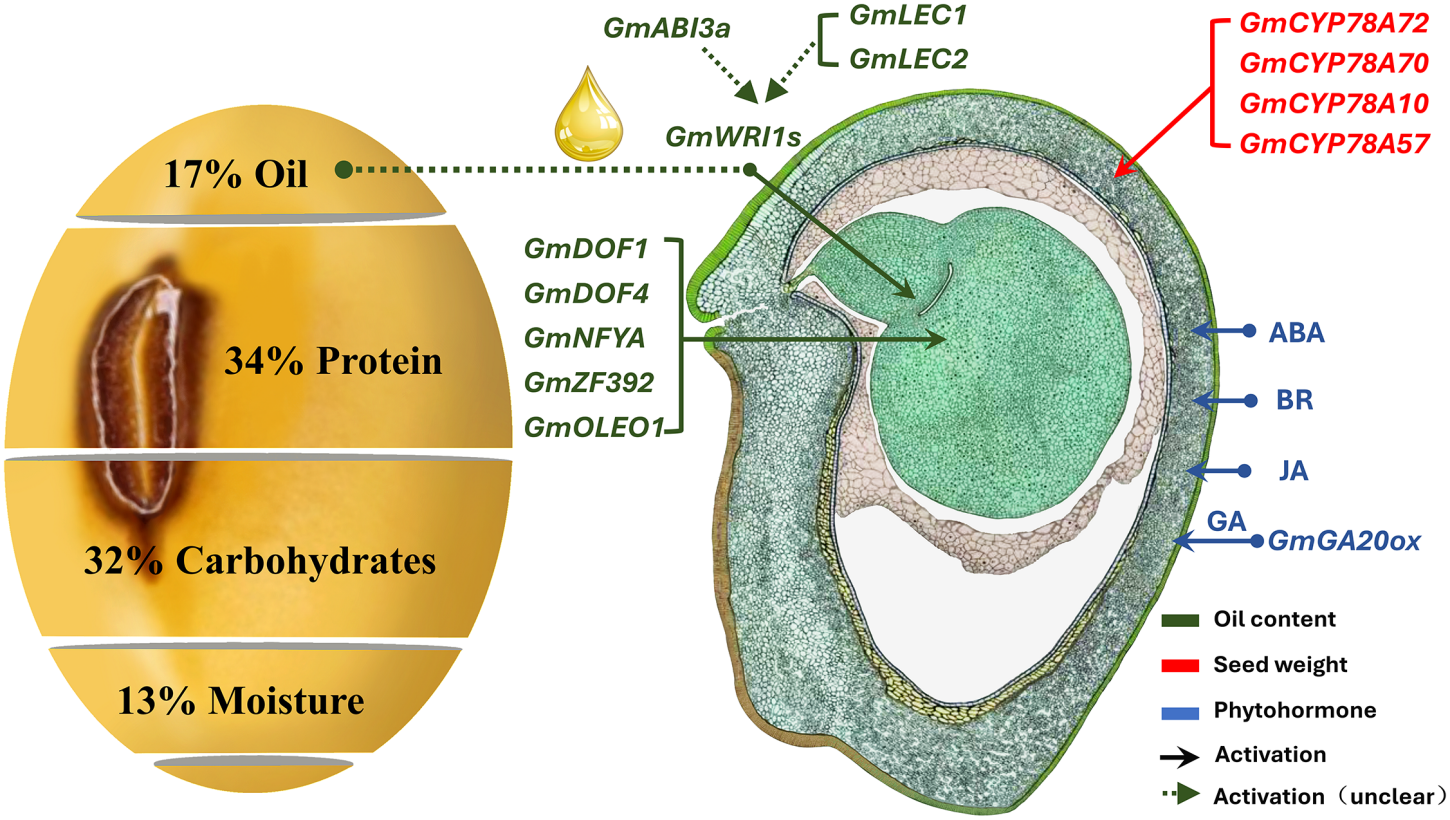
Schematic diagram of soybean seed weight (SW) regulation (Duan et al., 2023). The morphographic image was hand drawn by Sharon Lee Belkin (http://seedgenenetwork.net/soybean). The green box represents genes related to fatty acids in the process of SW regulation, and the red represents the process of seed development regulation.

Seed weight (SW) is a complex quantitative trait controlled by genetic and environmental factors (Shimada et al., 2016; Goettel et al., 2022). Heavier seeds can provide sufficient energy for seed germination and ensure the competitiveness of seedlings during their growth process (Nakabayashi et al., 2010). At present, hundreds of genes that participate in SW regulation have been reported, which are related to the seed coat, endosperm development, and hormone regulation (**Figure 1**). *GmGA20OX* encodes a member of the gibberellin 20 oxidase superfamily, which affects seed development through regulation of gibberellin synthesis (Tudzynski et al., 2002; Carmen et al., 2007). The expression level of *GmGA20OX* is positively correlated with SW; overexpression of *GmGA20OX* increased SW in *Arabidopsis* (Hu et al., 2023). *PP2C-1* encodes a phosphatase associated with the transcription factor *GmBZR1*, which is involved in brassinolide signaling (González-García et al., 2007; Singh et al., 2021), promoting the dephosphorylation of *GmBZR1* and enhancing the size of integument cells. The overexpression of *PP2C-1* can significantly increase the SW and seed size (Wang et al., 2018). Soybean cytochrome P450 (CYP450) positively regulates SW by controlling cell proliferation (Ohta et al., 2000; Guttikonda et al., 2010). Overexpression of the soybean CYP450 genes (*GmCYP78A5* and *GmCYP78A72*) considerably increases soybean SW (Du et al., 2017).

In addition to the positive regulation of soybean SW by the aforementioned genes, certain genes also play a negative role in regulating SW. For example, a knockout of the cell wall invertase inhibitor gene (*GIF1*) results in a considerable increase in cell wall invertase activity and SW (Kim and Hans, 2004). The ubiquitin receptor, *DA1*, negatively regulates seed size and SW by limiting the cell proliferation cycle (Mora et al., 2021). Furthermore, the overexpression of *DA1* interferes with mutations in rapeseed, leading to an increase in SW (Du et al., 2014). BIG SEEDS1 (*BS1*) encodes a plant-specific transcription regulatory factor, TIFY, which negatively regulates seed development by inhibiting cell proliferation (Yin et al., 2020). When soybean *BS1* homologous genes (*GmBA1* and *GmBA2*) were suppressed, SW increased. Many candidate genes related to SW have been identified; however, the low-density molecular makers mean that the candidate genes are less effective in crop improvement. In addition, the regulatory pathways of soybean SW have not yet formed a systematic molecular regulatory network.

The cultivated soybean (*Glycine max* (L.) Merrill) seeds contain approximately 17% oil (**Figure 1**). Soybean oil is stored mainly as fatty acids (FAs), triacylglycerols (TAGs), and tocopherols; there are five central FAs present in soybean seeds (Qin et al., 2021). The genes related to fatty acid synthesis, such as *DREBL* (Zhang et al., 2016), *DGAT* (Vyacheslav et al., 2010; Zhao et al., 2019), *Dof* (Zhang et al., 2014), and *WRINKLED1* (Vogel et al., 2019), regulate the development and maturation process of seeds and affect SW *via* the redistribution of storage substances (Che et al., 2016; Chen et al., 2020). WRI1 is a core transcription factor that regulates key enzymes for fatty acid biosynthesis; however, we lack an understanding of the molecular mechanisms underlying the regulation of SW by WRI1. Furthermore, the genetic mechanism for the synergistic regulation of SW and FAs remains poorly understood (Jaromír et al., 2015; Lu et al., 2016b; Hacisalihoglu et al., 2018). SW is regulated by numerous genes; therefore, the genetic complexity of quantitative traits controlled by multiple genes and their interaction with the environment make it difficult for these candidate genes to be directly used to guide molecular marker-assisted breeding. The present study can serve as a good reference for future studies on high-quality soybean breeding.

## 2 Materials and methods

### 2.1 Phenotyping of soybean germplasms

The 1210 soybean accessions from China were collected to obtain accurate phenotypic data for the GWAS. In 2020–2022, the plant materials were planted in three regions of China (Gongzhuling, Yantai, and Guangzhou), which represent typical climates (subtropical, tropical, and temperate zones). Seeds of different soybean accessions were dried after harvest to measure the SW and fatty acid content. The NIRSTM DS 2500 (FOSS Inc., Hillerod, Denmark) was used to measure the oil content of the soybean seeds. Five FAs (linolenic acid, stearic acid, oleic acid, palmitic acid, and linoleic acid) were determined by matrix-assisted laser desorption/ionization time-of-flight imaging mass spectrometry (MALDI-TOF IMS) (Bruker Daltonics, Bremen, Germany). Each calculation was repeated thrice. The correlation coefficients were calculated using the SPSS software 20.0 (SPSS Inc., Chicago, IL, USA).

### 2.2 Genotyping of soybean germplasms

Genomic DNA was extracted from young leaves of the natural population consisting of 1210 soybean accessions using the cetyltrimethylammonium bromide (CTAB) method (Porebski et al., 1997). For each accession, 6 μg of DNA was used to construct a sequencing library according to the manufacturer’s instructions. The same genotyping method was performed as previously described (Qin et al., 2021). The restriction endonuclease combination was *RsaI-HaeIII*. Single nucleotide polymorphism (SNP) molecular markers were used for the GWAS analysis.

### 2.3 GWAS and identification of candidate genes

Based on the phenotypic data of 1210 soybean germplasms, the SNP markers with a minor allele frequency (MAF) > 0.06 obtained from genotyping were included in the GWAS to analyze the association between genotype and phenotype datasets. Haploview 4.2 software was used for Manhattan map construction. We used plink^2^ software to calculate the linkage disequilibrium (LD) decay distances. The candidate genes were within the LD distance (8.9 Kb). We used 3,212,756 high-quality SNPs (MAF > 0.06) to perform GWAS for SW in the 1210 accessions. The fastlmmc model were used for association analyses. The threshold value for GWAS was set to –log(p) > 6.20. The significantly associated regions were verified from the aligned sequencing reads against the Williams 82 genome (*Glycine max* Wm82.a2.v1) with SAMtools ver. 0.1.18 (Li and Durbin, 2009). For each statistically significant SNP of interest, the annotated genes within 150 Kb around the SNP were considered as candidate genes (based on Williams 82).

### 2.4 Plasmid construction and plant transformation

The *GmWRI14-*like gene from the soybean cultivar 010a (approval number: 2012010) was cloned into the *BamHI-SacI* site of the plasmid *pTF101* named *pTF101-GmWRI14-3×Flag*, which was driven by the *CaMV35S* promoter. The target gene was terminated by the *NOS* terminator (**Supplementary Figure A1**). The recombinant plasmid was transformed into calli using the *Agrobacterium tumefaciens* strain (LBA4404). Transgenic soybean plants were generated as previously described (Holsters et al., 1978). Individual T_0_ plant lines were established in a greenhouse and three independent transgenic soybean lines with the same plasmid were harvested: *GmWRI14*-1, *GmWRI14*-2, and *GmWRI14*-3. Following a previous protocol (Bai et al., 2020), the novel CRISPR/Cas9 vector pGES201 was used for soybean genome editing. The *Cas9* gene driven by the *pM4* promoter (**Supplementary Figure A2**) and the Cas9 vector were gifted by Fujian Agriculture and Forestry University. The exon sequence of the *GmWRI14-*like gene in the soybean genome was determined using the CRISPR direct website (http://crispr.dbcls.jp/). We designed three target site sequences with a length of 20 bp (**Supplementary Figure A2**): WRI14-pM4-T1-F1/WRI14-pM4-T1-R1, WRI14-pM4-T2-F1/WRI14-pM4-T2-R1, and WRI14-pM4-T3-F1/WRI14-pM4-T3-R1. We simultaneously designed the CRISPR primers WRI14-CasF-Test/WRI14-CasR-Test. Three independent *gmwri14-*like soybean mutant lines containing the same plasmid were harvested: *gmwri14-*like*-1*, *gmwri14-*like*-2*, and *gmwri14-*like*-3*.

### 2.5 RNA extraction and sequencing

To explore the changes in differentially expressed genes (DEGs) and differential metabolic pathways in *GmWRI14-*like transgenic soybeans, RNA sequencing was performed on transgenic soybeans using a high-throughput Illumina HiSeq sequencing platform. Total RNA was extracted using the Eastep^®^ Super total RNA extraction kit (TaKaRa, USA). RNA quality was assessed using the Nanodrop 2000c (Thermo Scientific, Hudson, NH, US). RNA-seq library preparation and sequencing were performed as previously described (Wang et al., 2014). The 2^-ΔΔCt^ method was used to assess the fold change in gene expression (Livak and Schmittgen, 2001). To analyze the transduction pathways induced by the *GmWRI14-*like gene, the total RNA of each sample (*GmWRI14-*like transgenic soybeans, CK, *gmwri14-*like mutants) was isolated from the seeds for the RNA-sequencing; 2** g** of RNA was obtained from each plant sample. The RNA-sequencing depth was 10 ×. The bioinformatics software (https://www.bioinformatics.babraham.ac.uk/projects/trim_galore/) was used to improve data quality. In total, we obtained 41.66 Gb of clean data in this project. The statistical power of this experimental design, calculated in cluster Profiler Power, was equal to a *q* value ≤ 0.01. Gene Ontology (http://www.geneontology.org/) and Kyoto Encyclopedia of Genes and Genome (KEGG) statistical analysis (http://www.genome.jp/kegg/) were performed to assess the function of the DEGs.

### 2.6 Quantitative reverse transcription-polymerase chain reaction

After 10 days of podding, RNA was extracted from the seeds of the soybean lines (*GmWRI14-*like transgenic soybeans, *gmwri14-*like mutants, and control soybean lines). We then conducted 24 h sampling daily at 10, 11, 12, 13, and 14 days after podding (GT0, GT4, GT8, GT12, GT16, GT20, and GT24 soybean seeds, respectively) to extract total RNA. qRT-PCR was used to compare the overexpression of *GmWRI14-*like soybeans on different days and at different times to explore the molecular regulatory network related to the *GmWRI14-*like, *gmwri14-*like mutants, and control soybean plants involving the DEGs. cDNA synthesis was performed using a reverse transcription kit EZBiosciences (Omega. USA). Real-time fluorescence analysis was performed using a LightCycler (Roche, Rotkreuz, Switzerland) according to the manufacturer’s instructions (Livak and Schmittgen, 2001). All primers used are listed in the additional files (**Supplementary Table A1**).

### 2.7 Yeast two-hybrid assays and bimolecular fluorescence complementation

We performed a Y2H assay by screening the cDNA library constructed from the seed aged 15 days. The full-length GmWRI14-like protein was used as a bait, and we identified its putative partner using Y2H library screening. Y2H assays were performed as described previously (Xiaofei et al., 2017). The BiFC assay was performed as previously described (Mou et al., 2021).

## 3 Results

### 3.1 Phenotypic variation of SW between 2020 and 2022

**Figure 2:**
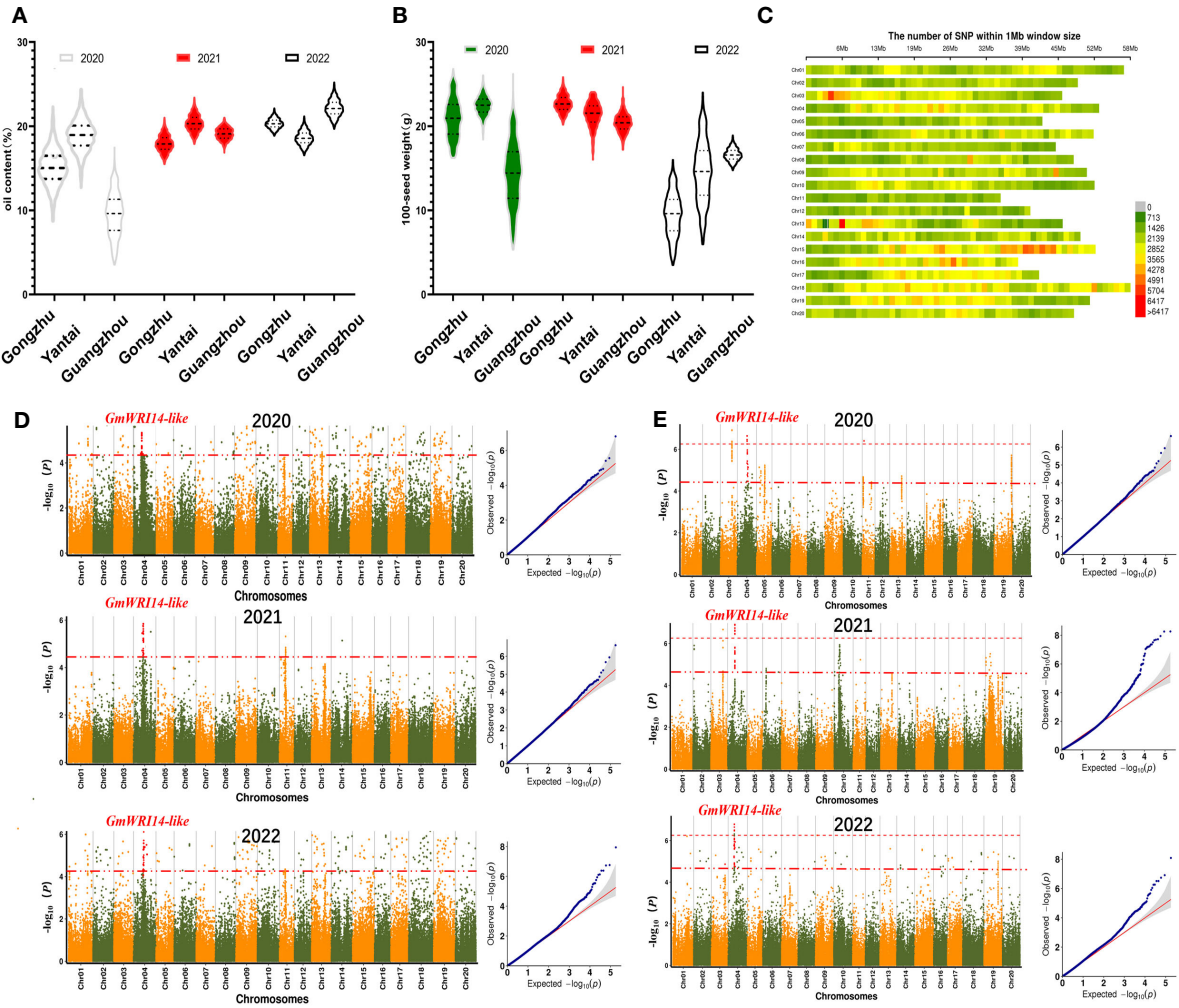
**(A)** Frequency distribution of oil content from the year 2020 to 2022. The 1210 soybean lines were grown in three different regions (Gongzhuling, Yantai, and Guangzhouling). Oil content conformed to normal distribution. In 2020, the oil content was the lowest in Gongzhuling (4.51–17.52%). **(B)** Frequency distribution of seed weight (SW) from the year 2020 to 2022. In 2022, the SW was the lowest in Guangzhou (3.85–14.26 g). **(C)** Distribution map of single nucleotide polymorphisms (SNPs) on different chromosomes. The abscissa is the length of the chromosomes. Each band represents one chromosome. The red area indicates that the number of SNPs exceeded 5704. The green area indicates that the number of SNPs was less than 1426. **(D)** From the year 2020 to 2022, *GmWRI14-*like was found to be a candidate gene for oil content. **(E)** From the year 2020 to 2022, genome-wide Manhattan plots of the associations of *GmWRI14-*like with SW.

Over 3 years of measurements, the variation in SW ranged from 5.43 g to 27.11 g in 2020 (standard deviation (SD): 7.6), 17.88 g to 26.89 g in 2021 (SD: 3.7), and 2.21 g to 23.59 g in 2022 (SD: 6.7). The variation in oil content ranged from 3.35% to 25.12% in 2020 (SD: 7.9), 15.52% to 25.19% in 2021 (SD: 2.4), and 17.33% to 27.83% in 2022 (SD: 2.6). The distribution of the SW was continuous, and both the SW and oil content conformed to normal distributions (**Figures 2A, B**).

### 3.2 A new candidate gene related to SW was discovered by GWAS

GWAS using the fastlmmc model was used to identify potential candidate genes associated with SW. The high-quality 3,212,756 SNP markers obtained by SLAF-Seq technology were used for GWAS for SW in the 1210 accessions. The SNP marker distributions are shown in **Figure 2C**. Manhattan maps were used to show the oil content (**Figure 2D**) and SW (**Figure 2E**) across different years; the threshold value was set at –log(p) > 6.20 (red). We identified one significant association loci for SW on chromosome 4 (**Figure 2E**), and we estimated the candidate region to be from 1.600 to 1.750 Mb (150 Kb) around the leading SNP in chromosome 4. This region contains 14 genes (**Supplementary Table A2**). According to Swissport annotation, these genes were annotated, such as gibberellin 20 oxidase, phosphatidylethanolamine-binding protein, and dof zinc finger protein, and had already been reported to affect SW (Wang et al., 2007; Lu et al., 2016a); therefore, we focused on *Glyma.04G116500.1*, which we labeled *GmWRI14-*like. It is speculated that *GmWRI14*-like belongs to the WRI1 family with two AP2/EREB domains (**Supplementary Figure A3**). The soybean line with the lowest SW (KH1088) harbored one SNP predicted to cause a gain in a stop codon, resulting in the premature termination of translation after 241 amino acids in the 710-amino acid *GmWRI14*-like protein.

### 3.3 Identification of the role of *GmWRI14*-like in controlling SW

**Figure 3:**
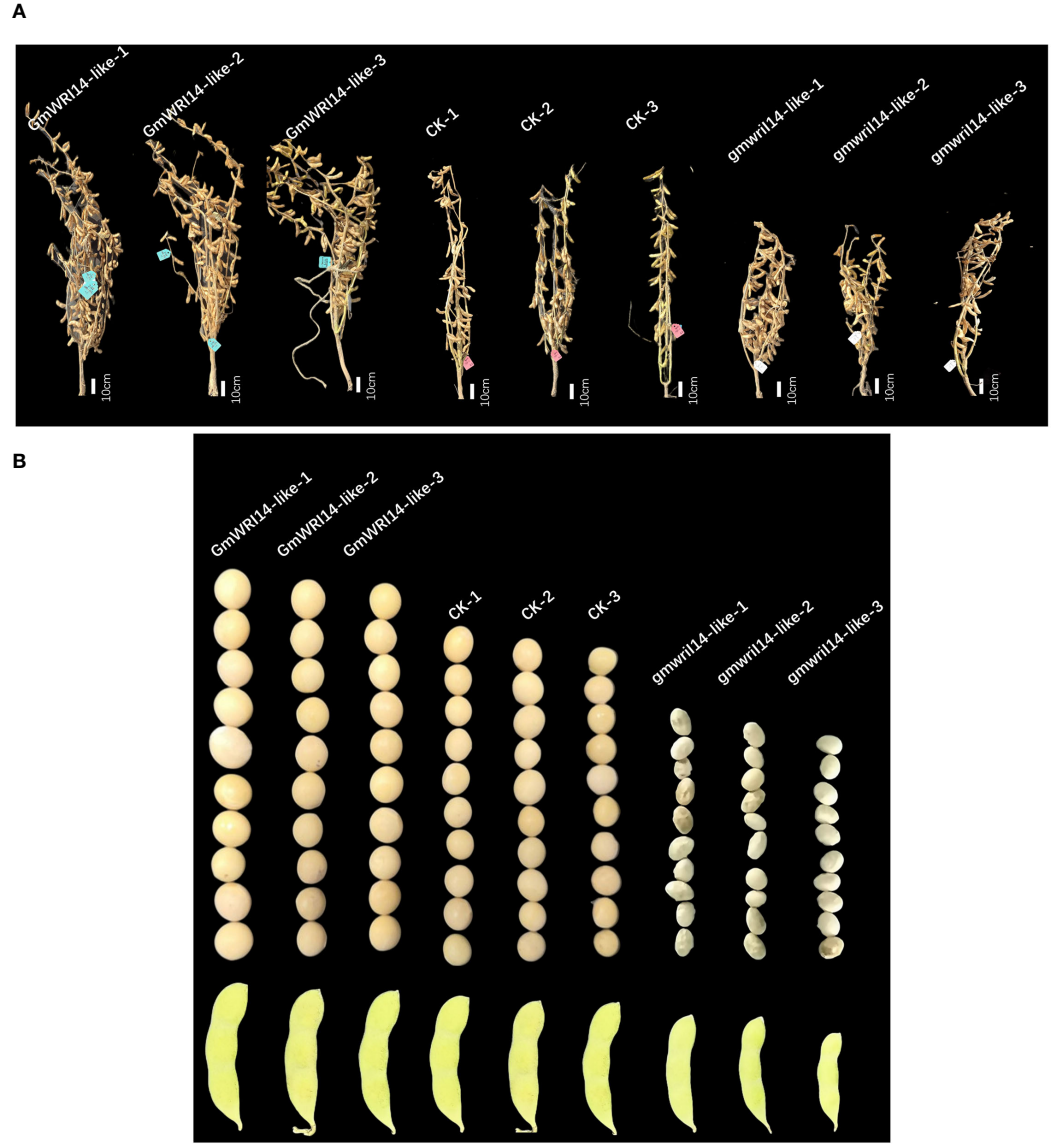
**(A)** *GmWRI14-*like overexpression soybean and *gmwri1-*like mutant phenotype. **(B)** The overexpression of the *GmWRI14-*like gene in soybeans resulted in an increase in seed size and SW, while the *gmwri14-*like mutant showed a decrease in SW and wrinkling. *GmWRI14-*like-1, 2, and 3: overexpressing *GmWRI14-*like lines, *gmwri1-*like*-*1, 2, and 3: different mutant lines.

**Figure 4:**
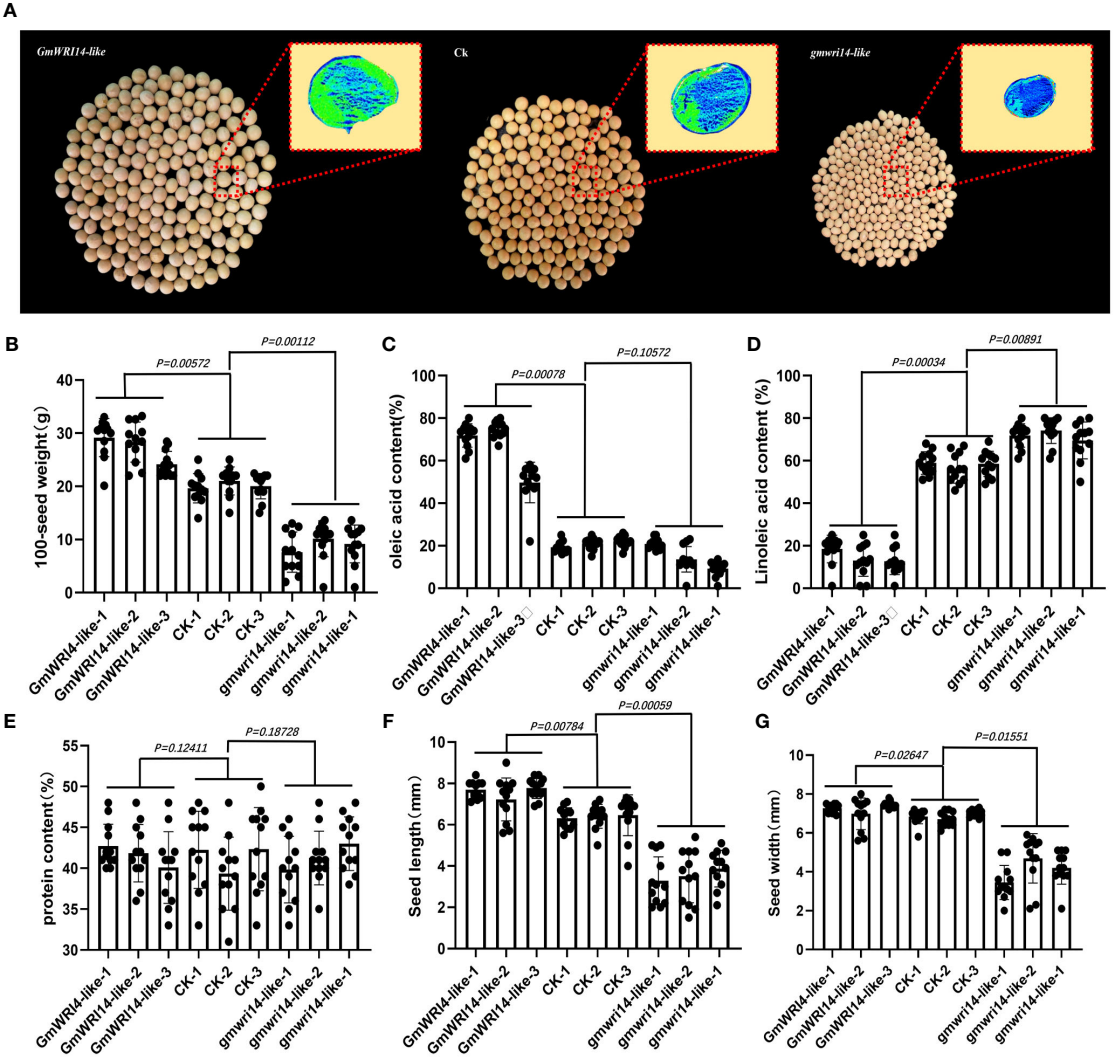
**(A)** Matrix-assisted laser desorption/ionization time-of-flight imaging mass spectrometry (MALDI-TOF IMS) analysis of the distribution of oil in the seeds. Survey results of 100 seed weights (SWs) **(B)**, The green area represents a higher density of oil distribution in the seeds, with a unit of 1 mm. Survey results of 100 seed weights (SWs), oleic acid **(C)**, linoleic acid **(D)**, protein content **(E)**, seed length **(F)**, and seed width **(G)** in transgenic *GmWRI14-*like soybeans, non-transgenic soybean recipients, and *gmwri14-lik*e mutant soybeans.

Using a stable soybean genetic transformation system, transgenic soybean plants overexpressing *GmWRI14-*like were successfully obtained (**Figure 3A**). Overexpression of *GmWRI14-*like in soybeans significantly increased seed size and SW compared to the wild-type soyabeans (*P<0.01*), resulting in severe bending of soybean branches (**Figure 3A**). The *gmwri14-*like soybean mutant was obtained using CRISPR/Cas9 genome editing technology (**Figure 3A**). Compared with the non-transgenic soybean control, the seed size of the *gmwri14-*like soybean mutant was significantly reduced (*P<0.01*), and the seed epidermis of the *gmwri14-*like soybean mutant exhibited wrinkling (**Figure 3B**). Overexpression of *GmWRI14-*like resulted in a 15.32% increase in seed length (*P<0.01*), whereas the seed length of the *gmwri14-*like mutant was reduced by 23% (**Figure 4F**). Furthermore, the width of the *GmWRI14*-like seeds was 14.29% greater than that of the CK. *GmWRI14-*like*-3* had the largest width, reaching 7.14 ± 0.03 mm, whereas the *gmwri14-*like mutant seeds showed a 18.23–23.57% decrease in width. The width of *GmWRI14-*like*-3* reached 6.34 ± 0.16 mm (**Figure 4G**). The phenotype of *GmWRI14-*like transgenic soybeans showed a significant difference in the color of the seed coat from the different soybean lines (*P<0.01*). The non-transgenic soybean seed coat was yellow, whereas the seed coat color of *GmWRI14-*like soybeans tended to be white. These results suggested that the *GmWRI14-*like gene regulates the cytochrome gene expression related to the seed coat.

### 3.4 Functional validation of GmWRI14-like by transgenic experiments

The oil content and distribution in *gmwri14-*like soybean mutants and overexpressing *GmWRI14-*like soybean seeds were detected by using MALDI-TOF IMS (**Figure 4A**). The GmWRI14-like overexpressing soybeans seeds showed a 16.14–19.92% increase in 100 seed weights (**Figure 4B**). The MALDI-TOF IMS results showed that, compared to control, the relative content of oleic acid in the *gmwri1-*like mutant significantly decreased from 22.12% to 15.14% (*P<0.01*), whereas the oleic acid content in the *GmWRI14-*like overexpressing soybeans increased from 22.12% to 74.31% (**Figure 4C**). The relative content of linoleic acid in the *gmwri1-*like mutant increased by 8.2% (*P<0.01*), whereas the linoleic acid content in *GmWRI14-*like soybeans decreased from 49.31% to 18.42% (**Figure 4D**). There was no significant difference in protein content among the *GmWRI14-*like soybean, *gmwri14-*like mutant, and non-transgenic soybean (**Figure 4E**). These results indicate that the *GmWRI14-*like gene does not affect the protein content in soybean seeds.

### 3.5 Transcriptome analysis of the *GmWRI14-*like overexpression soybean

**Figure 5:**
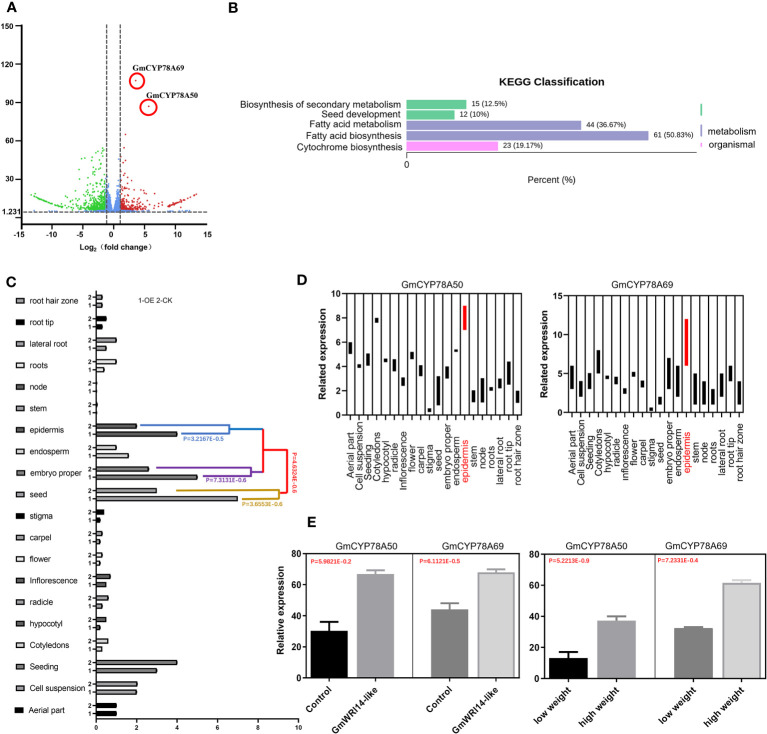
Differentially expressed genes (DEGs) related to seed weight (SW) in the *GmWRI14*-like transgenic soybeans. **(A)** DEGs of the *GmWRI14*-like transgenic soybean; the down-regulated differential genes (blue), and up-regulated genes (red). **(B)** KEGG pathway enrichment analysis. The differential genes were enriched in four signaling pathways, of which the signaling pathways with many enriched genes and related to seed development mainly included fatty acid biosynthesis (50.83%), fatty acid metabolism (36.37%), and cyto-chrome biosynthesis (19.17%). **(C)** *GmWRI14-*like expression in different tissues of the soybean. The *GmWRI14-*like expression increased in specific tissues (epidermis, embryo, and seed) of the *GmWRI14-*like transgenic soybean. **(D)** The *GmCYP78A50* and *GmCYP78A69* expression in different tissues of *GmWRI14-*like transgenic soybeans. **(E)** The *GmCYP78A50* and *GmCYP78A69* expression in the control soybean and *GmWRI14-*like transgenic soybean.

RNA-seq was performed on the transgenic and control groups (three replicates), resulting in more than 5.1 × 10^7^ clean reads; 2101 DEGs were screened through transcriptome analysis. The differential expression gene analysis (DEGVP) showed that two genes related to cytochrome metabolism were differentially expressed (more than 2.5 times), namely, *GmCYP78A50* (*Glyma.07G052300*) and *GmCYP78A69* (*Glyma.16G021200*) (**Figure 5A**). We analyzed the functions of the target genes and potential target genes using the KEGG pathway (**Figure 5B**), which showed that the DEGs were enriched in three signaling pathways related to seed development, including fatty acid biosynthesis (50.83%), fatty acid metabolism (36.37%), and cytochrome biosynthesis (19.17%) (**Figure 5B**).

To explore the role of *GmWRI14-*like in more detail, we measured the expression level of the *GmWRI14-*like gene in different soybean tissues (root, stem, leaf, and seed) using qRT-PCR and found that the *GmWRI14-*like gene had the highest expression level in soybean seeds (**Figure 5C**). The qRT-PCR results showed that *GmWRI14-*like gene expression was highly tissue-specific in soybean plants (**Figure 5C**). We used the *lectin* gene (Gene ID: 100775957) as a reference gene and detected the expression levels of *GmCYP78A50* and *GmCYP78A69* in different transgenic soybean tissues using qRT-PCR, which suggested that the expression levels of *GmCYP78A50* and *GmCYP78A69* were the highest in the epidermis of transgenic soybean seeds (**Figure 5D**). *GmCYP78A50* and *GmCYP78A69* showed an increased expression level in the transgenic plants than in the control plants (**Figure 5E**). Based on the RNA-seq and qRT-PCR results, we speculate that the *GmWRI14-*like gene regulates the expression of *GmCYP78A50* and *GmCYP78A69* in soybean seeds.

### 3.6 GmWRI14-like interacts with GmCYP78A50 and GmCYP78A69

**Figure 6:**
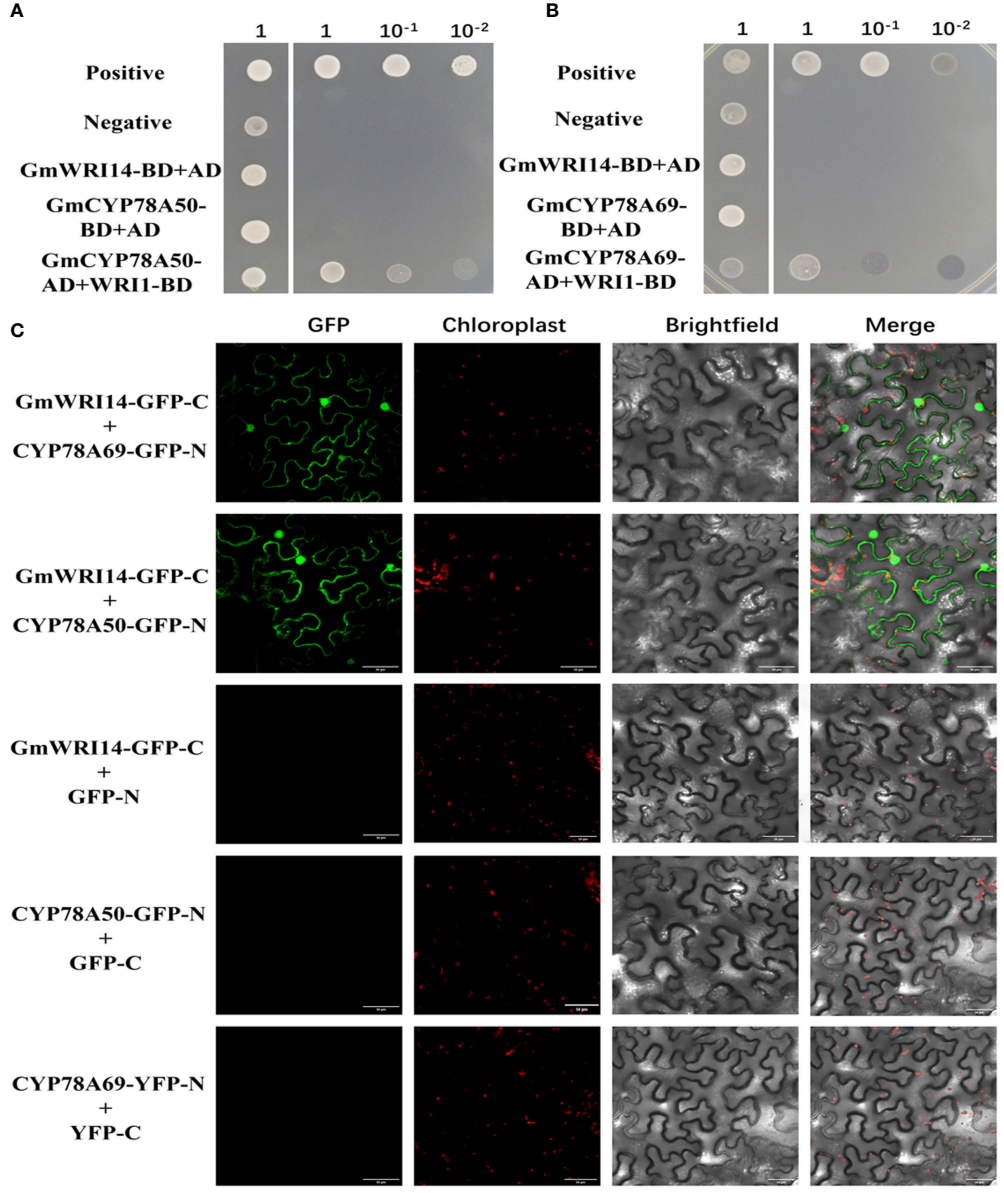
GmCYP78A69/GmCYP78A50 interacted with GmWRI114-like. **(A)** The interaction between GmCYP78A50 and GmWRI114-like was confirmed in the yeast two-hybrid (Y2H) assay. **(B)** The interaction between GmCYP78A69 and GmWRI114-like was confirmed in the yeast two-hybrid (Y2H) assay. Positive control: pGADT7-T + pGBKT7-53; negative control: pGADT7-T + pGBKT7-lam. GmWRI114-like-BD, GmWRI114-like fused to GAL4-BD; AD, GAL4-AD vectors without insertion. **(C)** The interaction between GmCYP78A69/GmCYP78A50 and GmWRI114-like was confirmed with bimolecular fluorescence complementation (BiFC).

We found 140 unique cDNA clones using Y2H. Interestingly, among the interacting proteins, GmCYP78A50 (*Glyma.07G052300*) and GmCYP78A69 (*Glyma.16G021200*) were identified multiple times. We further confirmed the interaction between GmWRI14-like and GmCYP78A50/GmCYP78A69 by point-to-point Y2H. First, we used GmWRI14*-*like bait and GmCYP78A50/GmCYP78A69 as prey (i.e., fused with GAL4-BD and GAL4-AD, respectively) and confirmed the interaction through Y2H. As an additional control, we fused another coiled coil domain protein (structural maintenance of chromosome 5) with GAL4-AD and another AP2 transcription factor (dehydration response element binding protein 2A) with GAL4-8D and tested their interactions with WRI1-BD and GmCYP78A50/GmCYP78A69-AD, respectively. Only when GmWRI14-like-BD binds to GmCYP78A69/GmCYP78A50-AD clones can they grow on selective media, indicating that the interaction is specific (**Figures 6A, B**). In addition, the interaction between GmWRI14-like and GmCYP78A50/GmCYP78A69 was further confirmed by BiFC (**Figure 6C**). Therefore, these results show that the GmWRI14-like protein can interact with CYP78A50/GmCYP78A69.

## 4 Discussion

Cultivated soybeans (*Glycine max* L. Merr.) are domesticated from annual wild progenitors (*Glycine soja*) (Kuroda et al., 2009). There are significant differences in the SW and oil content between cultivated and wild soybeans (Dong et al., 2021). The composition and ratio of soybean FAs determine the quality of the soybean oil, which is mainly composed of five FAs (Rahman et al., 2003; Harris et al., 2008; Martin et al., 2008). SW and oil content are highly correlated agronomical traits; however, there is currently a lack of a molecular basis for the synergistic regulation of SW and oil content (Duan et al., 2022; Duan et al., 2023). The candidate genes that specifically regulate SW have no significant impact on seed oil content (Fasoula et al., 2004; Fasoula and Boerma, 2007; Ramya et al., 2010). Therefore, exploring new candidate genes associated with oil content and SW through GWAS would provide novel ideas for high-yield breeding (Surabhi et al., 2009).

Over recent decades, some genes have been shown to affect SW (Chen et al., 2020), including oleate dehydrogenase (FAD2), which is a key enzyme in linoleic acid synthesis (Pham et al., 2010). Soybean mutants with a low linoleic acid content were obtained through targeted editing of *GmFAD2-1A*, resulting in a 2.62% increase in yield and a significantly higher SW than that of the control group (Hoshino et al., 2010). Linoleic acid dehydrogenase (FAD3) catalyzes the conversion of linoleic acid to linolenic acid (Dominguez et al., 2010). The overexpression of *GmFAD3* induces linolenic acid accumulation and jasmonic acid accumulation (Parida et al., 2017), which results in larger and heavier seeds (Bocianowski and Bartkowiak-Broda, 2012). DGAT is a rate-limiting enzyme in triacylglycerol synthesis, and the preference for the different types of DGAT selection could affect the SW (Charles et al., 2011). In a previous study, soybean *GmDGAT2D* was shown to prioritize the use of linoleic acid acyl chains as substrates; the linoleic acid content in *OE-GmDGAT2D Arabidopsis* was significantly higher than that of the control, whereas the thousand SW of *OE-GmDGAT2D Arabidopsis* decreased (Turchetto-Zolet et al., 2011). *GmDGAT1A* prioritizes the use of linolenic acid acyl chains as substrates, and *GmDGAT1A* reduces linoleic acid content in *Arabidopsis*, resulting in an increase in the thousand SW (Zhao et al., 2019).

The soybean transcription factors (TFs) Dof (*GmDof4* and *GmDofll)* increase the oil content of *Arabidopsis* and the SW (Zhang et al., 2014). The soybean DREB transcription factor *GmDREBL* plays an important role in seed oil accumulation. Wang et al. (2007) overexpressed *GmDREBL* in *Arabidopsis*, which resulted in a significant increase in the SW (Wang et al., 2007).

Plant cytochrome P450 is a monooxygenase encoded by a super gene family (Bernard et al., 2004). This enzyme usually combines with the membrane system of organelles and participates in many metabolic processes, including various fatty acid conjugates, plant hormones, secondary metabolites, etc. (Fissithaler et al., 1999). Some studies have shown that the CYP83 family of genes *CYP83A1* and *CYP83B1* play important roles in the dynamic balance of auxin and the growth and development of *Arabidopsis* (Liu et al., 2012). The *CYP78A5* gene in *Arabidopsis* affects cell proliferation and organ size. Soybean *CYP78A10* and *Arabidopsis CYP78A* have similar functions, mainly in the regulation of seed size, SW, and pod number, suggesting that this gene may also regulate seed development (Bernard et al., 2004). Studies have also indicated that *GmCYP78A72* regulates flower and seed development in soybeans; *Arabidopsis thaliana* lines overexpressing the *GmCYP78A72* gene exhibit increased leaf, petal, seed, and carpel traits. The overexpression of *GmCYP78A72* in soybeans can also cause soybean seeds to grow larger (Dai et al., 2016).

**Figure 7:**
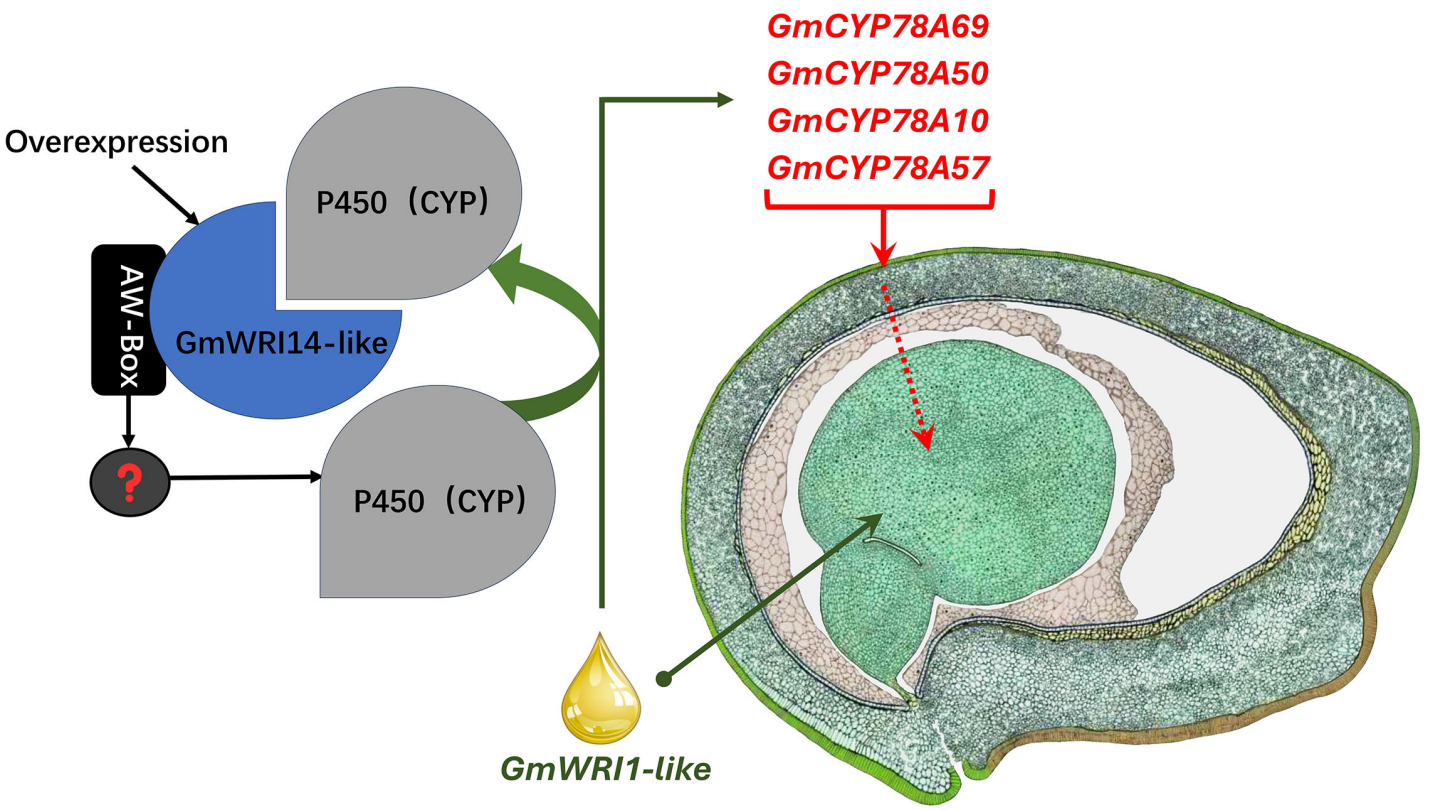
The model depicting transcriptional control by *GmWRI14*-like. The right image was hand drawn by Sharon Lee Belkin (http://seedgenenetwork.net/soybean).

Although several TFs, such as WRI1, FUS3, and LEC1, play important roles in seed development (Liu et al., 2010), their mechanism is yet to be clarified, especially in soybean seeds (Duan et al., 2023). WRI1 is an important transcription factor that regulates the distribution of carbon elements to the synthesis of FAs in seeds (Baud et al., 2007; Vogel et al., 2019). WRI1 not only increases the FA content in the seed, but also alters the SW through cross-family interactions with multiple transcription, post-transcription, and post-translational regulators (Maeo et al., 2010; Dahee and Suh, 2015). Therefore, this gene has the potential to improve the plant yield. In the *Arabidopsis* mutant, *Atwri1*, the weight of 1000 seeds decreased, total seed oil decreased, and linoleic acid content significantly increased by 15% (Baud et al., 2007). After expressing the exogenous gene *AtWRI1* in soybeans, the total oil content increased, and linoleic acid content decreased from 52.4% to 4.6% (Vogel et al., 2019). WRI1 can also directly or indirectly affect the activity of *Dof11*, *ACBP*, *PDCT*, and *FAD2* by binding to AW-box cis-acting elements (Zhou et al., 2020). Similar results were reported in our research; we found that the overexpression of *GmWRI14*-like not only increased the FA content but also significantly increased the SW. However, the molecular mechanism of *GmWRI14*-like is unclear. RNA-seq and qRT-PCR results indicated that *GmCYP78A50* and *GmCYP78A69* expression is increased in the transgenic plants compared to the control plants. We further confirmed the interaction between GmWRI14-like and GmCYP78A50/GmCYP78A69 by point-to-point Y2H and BiFC. Taken together, our data indicate that *GmWRI14*-like not only regulates the expression level of *GmCYP78A50* and *GmCYP78A69*, but also interacts with GmCYP78A50 and GmCYP78A69, which has not been reported in previous studies. According to our results, it is reasonable to speculate that the *GmWRI14-*like gene can directly or indirectly regulate *GmCYP78A50* and *GmCYP78A69* expression (depicted as “?” in **Figure 7**), and that GmCYP78A50 and GmCYP78A69 protein interaction with GmWRI14-like regulate its transcription in soybean seeds. Future studies should assess the precise function of *GmCYP78A50* and *GmCYP78A69* in soybean seeds, and their effect on *GmWRI14*-like transcription.

## 5 Conclusions

In this study, we identified a novel candidate gene, *GmWRI14-*like, that is associated with soyabean SW using a GWAS. Overexpression of the *GmWRI14-*like gene not only increased the total FA content of the seeds, but also increased the SW. RNA-seq and qRT-PCR results indicated that the upregulation of *GmCYP78A50* and *GmCYP78A69* is triggered by the transcription factor *GmWRI14-*like. Furthermore, the protein interaction between GmCYP78A69/GmCYP78A50 and GmWRI14-like was confirmed by using Y2H and BiFC.

## Data availability statement

The original contributions presented in the study are publicly available. This data can be found here: https://www.ncbi.nlm.nih.gov/ PRJNA608146 and PRJNA681350.

## Author contributions

DQ: Data curation, Investigation, Software, Writing – original draft, Writing – review & editing. LD: Writing – original draft, Data curation. LJ: Conceptualization, Writing – original draft, Formal Analysis, Methodology. XL: Conceptualization, Investigation, Software, Writing – original draft. PC: Formal Analysis, Funding acquisition, Project administration, Validation, Writing – review & editing. BL: Writing – review & editing, Supervision. GY: Writing – original draft, Investigation, Supervision.

## Supplementary material

Supplementary Figure 3 Multiple alignment. *GmWRI14*-like contained two (AP2/EREB) DNA-binding domains. The homology of the amino acid sequences between *GmWRI14*-like and *AtWRI1* (Gene ID: 824599) is 62.34%.

Supplementary Table 1 Primer sequence for qRT-PCR.
