# Supplementary material for: Genome-wide association study and RNA-seq identifies GmWRI1-like transcription factor related to the seed weight in soybean
Source: Front Plant Sci. 2023 Nov 17;14:1268511. doi: 10.3389/fpls.2023.1268511 (PMC10691256; doi:10.3389/fpls.2023.1268511)
Supplement: Supplementary Figure 3 — Multiple alignment. GmWRI14-like contained two (AP2/EREB) DNA-binding domains. The homology of the amino acid sequences between GmWRI14-like and AtWRI1 (Gene ID: 824599) is 62.34%. [file DataSheet_1.zip › Supplementary Files/Supplementary Files Figure A1-3.pptx]

## Slide 1
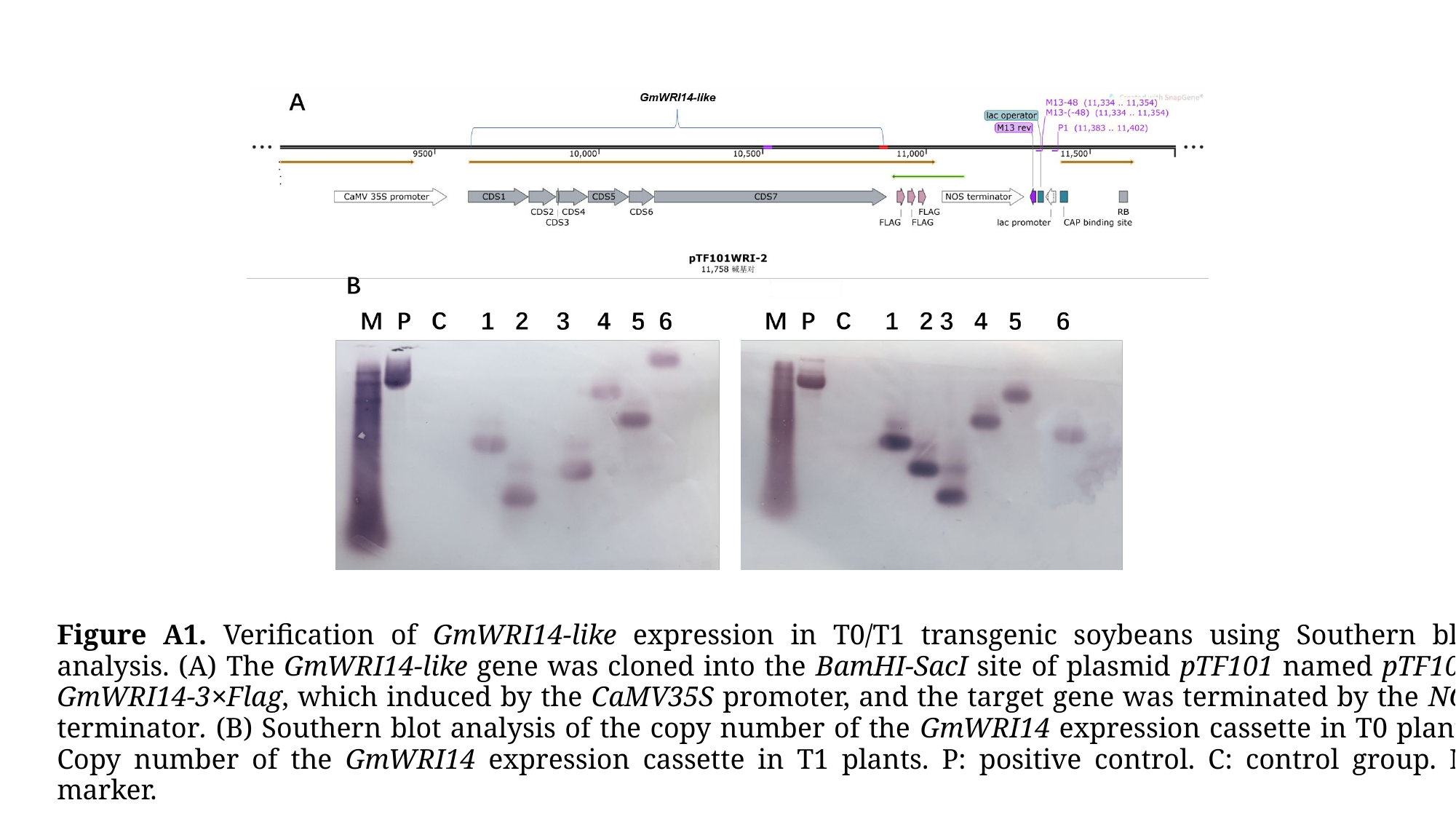

Figure A1. Verification of GmWRI14-like expression in T0/T1 transgenic soybeans using Southern blot analysis. (A) The GmWRI14-like gene was cloned into the BamHI-SacI site of plasmid pTF101 named pTF101- GmWRI14-3×Flag, which induced by the CaMV35S promoter, and the target gene was terminated by the NOS terminator. (B) Southern blot analysis of the copy number of the GmWRI14 expression cassette in T0 plants. Copy number of the GmWRI14 expression cassette in T1 plants. P: positive control. C: control group. M: marker.

## Slide 2
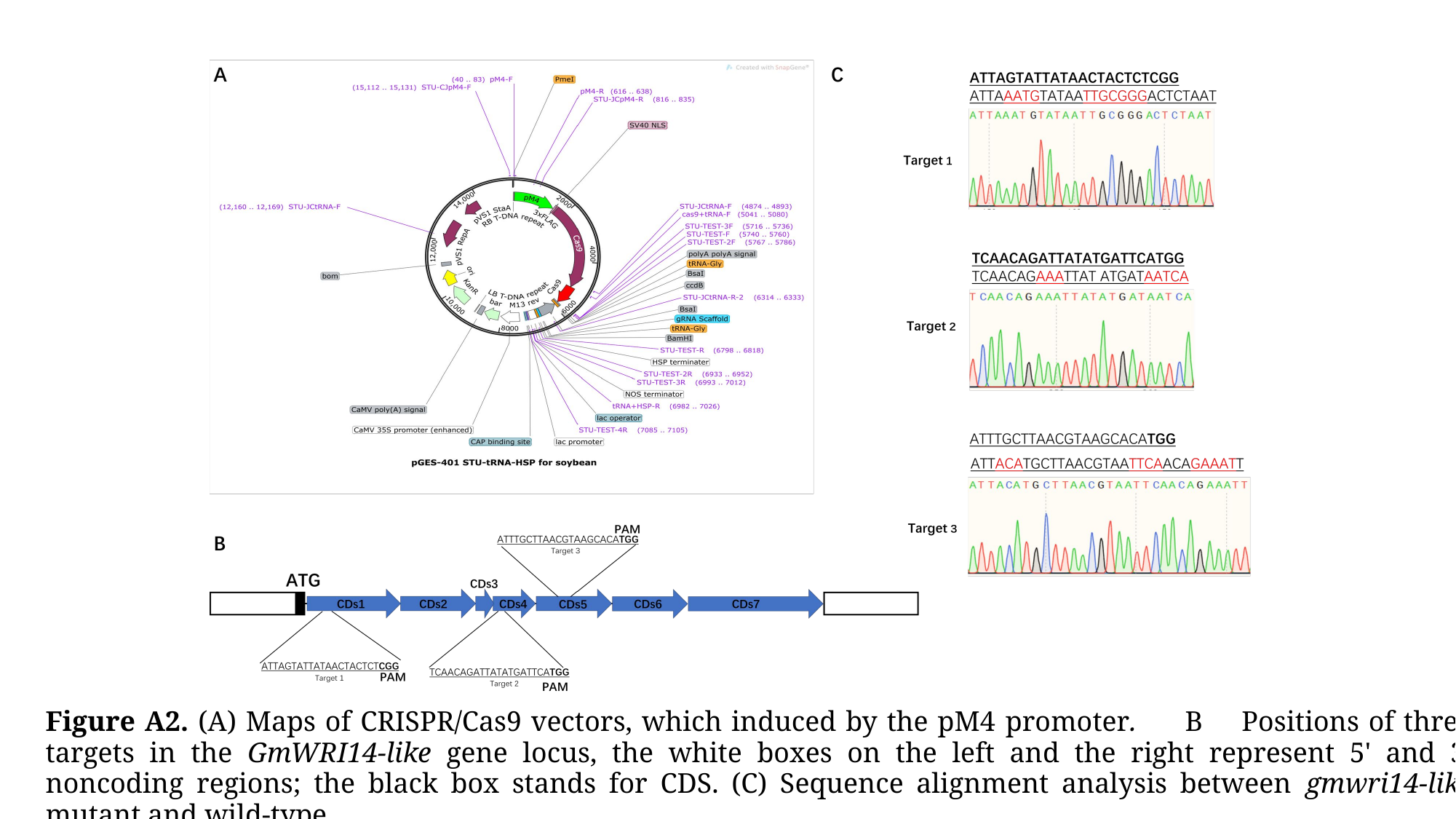

Figure A2. (A) Maps of CRISPR/Cas9 vectors, which induced by the pM4 promoter. （B）Positions of three targets in the GmWRI14-like gene locus, the white boxes on the left and the right represent 5' and 3' noncoding regions; the black box stands for CDS. (C) Sequence alignment analysis between gmwri14-like mutant and wild-type

## Slide 3
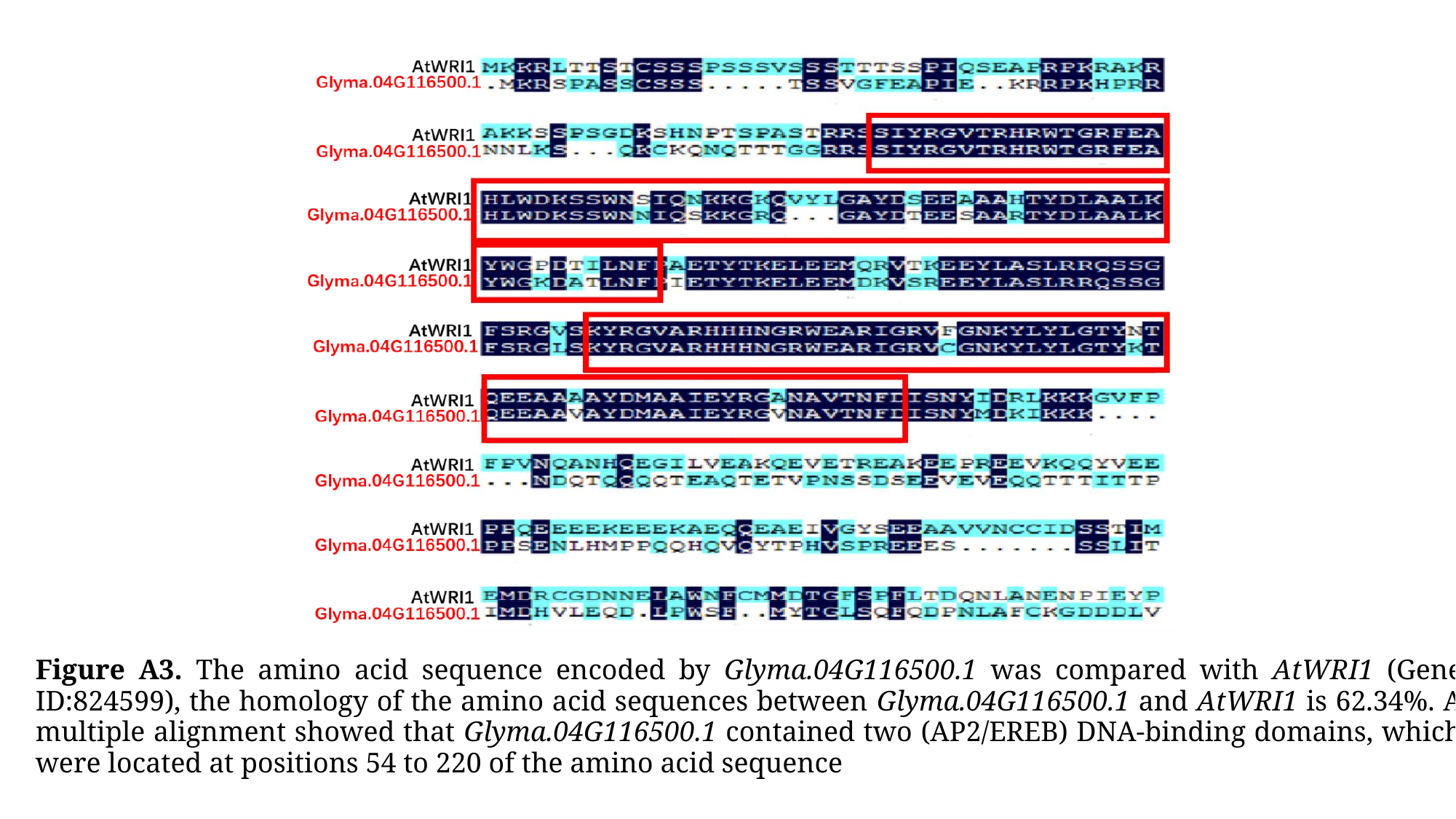

Figure A3. The amino acid sequence encoded by Glyma.04G116500.1 was compared with AtWRI1 (Gene ID:824599), the homology of the amino acid sequences between Glyma.04G116500.1 and AtWRI1 is 62.34%. A multiple alignment showed that Glyma.04G116500.1 contained two (AP2/EREB) DNA-binding domains, which were located at positions 54 to 220 of the amino acid sequence
