# Supplementary material for: Genome-wide association study and RNA-seq identifies GmWRI1-like transcription factor related to the seed weight in soybean
Source: Front Plant Sci. 2023 Nov 17;14:1268511. doi: 10.3389/fpls.2023.1268511 (PMC10691256; doi:10.3389/fpls.2023.1268511)
Supplement: Supplementary Figure 3 — Multiple alignment. GmWRI14-like contained two (AP2/EREB) DNA-binding domains. The homology of the amino acid sequences between GmWRI14-like and AtWRI1 (Gene ID: 824599) is 62.34%. [file DataSheet_1.zip › Supplementary Files/Supplementary Files Table-A1.docx]

| Primer name | Sequence (5^,^-3^,^) |
| --- | --- |
| Lectin- P1 | 5′-GCACTTAAGATACTCTAGGTAC-3′ |
| Lectin- P2 | 5′-CCACCTCCCTACTATCCATT-3′ |
| GmWRI14-like-p3 | 5′-TTGCCTGTCTAGATCCACAGCTGGTACCGAT-3′ |
| GmWRI14-like-p4 | 5′-TTGTGACCTCGACCTATTGGCGTTACCAATT-3′ |
| GmCYP78A50-P5 | 5′-TCCACCTTCATGCTTTGCCGTTACAAGACTCTACT-3′ |
| GmCYP78A50-P6 | 5′-TGGATCCGGCTCGCTACCTTCAACTTACTTAACTG-3′ |
| GmCYP78A69-P7 | 5′-TATGTATCTCCATTCCTCTTC-3′ |
| GmCYP78A69-P8 | 5′-AATCTTCGTTCTGCTGTT-3′ |
| WRI14-pM4-T1-F1 | 5′-TGGTCTCGTGCAGATGATGAACAAGAAGATGCGTTTTAGAGCTAGAAATAGC-3′ |
| WRI14-pM4-T1-R1 | 5′-TGGTCTCgcttCAAACCTCCtgcaccagccgggaatcgaa-3′ |
| WRI14-pM4-T2-F1 | 5′-TGGTCTCGAAACTCTTGCTCTGAATGTTGTTCTGCACCAGCCGGGAATCGAA-3′ |
| WRI14-pM4-T2-R1 | 5′-TGGTCTCGGAAGCTCACCTAGTTTTAGAGCTAGAAATAGC-3′ |
| WRI14-pM4-T3-F1 | 5′-TTACGCCAAGCTTGCATGCCGAGGACCTAACAGACTGGCGAAC-3′ |
| WRI14-pM4-T3-R1 | 5′-GTCTTCTGAAaTCGTCGACCTGCAGGGATCTAGTAACATAGATGACACCGCGC-3′ |
| WRI14-pM4-CasF-Test | 5′-GAGGGATTGTAGTTCTGTTG-3′ |
| WRI14-pM4-CasF-Test | 5′-CTATGGCCCATTGGTTGCTC-3′ |
