# Supplementary material for: Genome-wide association study and RNA-seq identifies GmWRI1-like transcription factor related to the seed weight in soybean
Source: Front Plant Sci. 2023 Nov 17;14:1268511. doi: 10.3389/fpls.2023.1268511 (PMC10691256; doi:10.3389/fpls.2023.1268511)
Supplement: Supplementary Figure 3 — Multiple alignment. GmWRI14-like contained two (AP2/EREB) DNA-binding domains. The homology of the amino acid sequences between GmWRI14-like and AtWRI1 (Gene ID: 824599) is 62.34%. [file DataSheet_1.zip › Supplementary Files/Supplementary Files Table-A2.docx]

Table.A2. Predicted genes in the target 150 kb genomic region in the reference genome of Williams82

| Gene | Predicted Function |  | | Contribution rate | |
| --- | --- | --- | --- | --- | --- |
| *Glyma.04G116100.1* | gibberellin 20-oxidase |  | | 0.60 | |
| *Glyma.04G116200.1* | phosphatidylethanolamine-binding protein |  | | 0.62 |  |
| *Glyma.04G116500.1* | WRI1 protein family | |  | 0.63 |  |
| *Glyma.04G116300.1* | Glycine max uncharacterized, mRNA |  | | 0.68 |  |
| *Glyma.04G116400.1* | Glycine max probable transcriptional regulatory protein, mRNA | |  | 0.61 |  |
| *Glyma.04G116600.1* | Glycine max ATP carrier protein 1, chloroplastic-like, mRNA | |  | 0.62 |  |
| *Glyma.04G116700.1* | Glycine max VIN3-like protein, mRNA | |  | 0.60 |  |
| *Glyma.04G116800.1* | Glycine max uncharacterized, mRNA | |  | 0.58 |  |
| *Glyma.04G116900.1* | Dof zinc finger protein | |  | 0.60 |  |
| *Glyma.04G117000.1* | Glycine max DNA replication complex BAG protein，transcript variant 2, mRNA， | |  | 0.57 |  |
| *Glyma.04G117100.1* | Glycine max protein S-acyltransferase 24-like, misc_RNA | |  | 0.52 |  |
| *Glyma.04G117200.1* | Glycine max uncharacterized, mRNA | |  | 0.47 |  |
| *Glyma.04G117300.1* | Glycine max tRNA methyltransferase 10 homolog A-like (LOC100779099), mRNA | |  | 0.60 |  |
| *Glyma.04G117400.1* | Glycine max uncharacterized, mRNA | |  | 0.48 |  |
